# Gut dysbiosis and brain microhemorrhages in young vs. aged mice with chronic kidney disease

**DOI:** 10.1038/s41598-025-17751-2

**Published:** 2025-09-25

**Authors:** Yitong Zhao, Tiffany Tran, Chuo Fang, Annlia Paganini-Hill, Madison Dulkanchainun, Emily Mai, Lucy Eprem, David Cribbs, Mark Fisher, Wei Ling Lau

**Affiliations:** 1 Department of Medicine, Division of Nephrology, University of California, Irvine, CA USA; 2 Department of Neurology, University of California, Irvine, CA USA; 3 Beckman Laser Institute and Medical Clinic, University of California, Irvine, CA USA; 4 Institute for Memory Impairments and Neurological Disorders, University of California, Irvine, CA USA; 5 Department of Pathology & Laboratory Medicine, University of California, Irvine, CA USA

**Keywords:** Chronic kidney disease, Gut dysbiosis, Cerebral microhemorrhages, Aging., Chronic kidney disease, Dysbiosis, Blood-brain barrier, Neural ageing

## Abstract

Intestinal dysbiosis and gut-derived toxins in chronic kidney diseases (CKD) are associated with vascular injury. This study examined the relationship between gut dysbiosis and cerebral microhemorrhages (CMH) in young and aged CKD mice (3 vs. 16 months of age) in both sexes. CKD was induced in C57BL/6J mice using a nephrotoxic adenine diet. Serum creatinine, trimethylamine N-oxide (TMAO), indoxyl sulfate (IS) and p-cresyl sulfate (pCS) were measured. CMH was quantified via brain histology, and gut microbial sequencing was analyzed from fecal pellets. Creatinine and uremic toxins were elevated in both young and aged CKD mice compared with controls, and microbial populations were altered by age, sex and CKD status. Age was the most significant factor in microbial variance, with higher levels of IS and pCS in aged CKD mice. Aged male mice had significantly higher creatinine, TMAO and IS than aged females. Males had higher CMH counts than females, and aged CKD males had the highest CMH burden. Age modified the relationship between uremic toxins and CMH burden, with creatinine, TMAO and IS correlating with increased CMH in aged animals. In conclusion, gut dysbiosis in CKD is modulated by sex and age, and gut-derived uremic toxins including TMAO and IS may contribute to vascular injury and CMH development.

## Introduction

Gut dysbiosis occurs in several chronic disease states including chronic kidney disease (CKD)^1–3^. Prior studies have described pathways of communication in the kidney-gut-brain axis^4^. Chronic inflammation and oxidative stress in CKD with increased reactive oxygen species (ROS) can alter intestinal permeability via degradation of the mucus lining^5^. Retained urea and other waste products alter microbial populations in the gut lumen, ultimately fostering production of toxins which enter the bloodstream^3,6^. Evidence to date supports a role for these gut-derived toxins – such as indoxyl sulfate (IS), p-cresyl sulfate (pCS) and trimethylamine N-oxide (TMAO) – in both vascular injury and CKD progression^7–9^.

Cerebral small vessel disease is increased in CKD and includes microinfarcts, white matter atrophy, lacunes, arteriosclerosis and cerebral microhemorrhages (CMH)^10^. Mechanisms underlying this association are still under investigation and include CKD-specific factors such as uremic toxins, salt retention, and vascular calcification^10,11^.

We previously reported that CKD predicts cerebral small vessel disease even in the oldest-old adult population aged ≥ 90 years^12^and that in vitro treatment with a combination of uremic toxins and lipopolysaccharide disrupts the brain endothelial monolayer^11^. The present study was undertaken to further investigate the kidney-gut-brain axis in uremic CMH formation in young vs. aged animals.

## Results

### Brain microhemorrhage formation in CKD

**Fig. 1:**
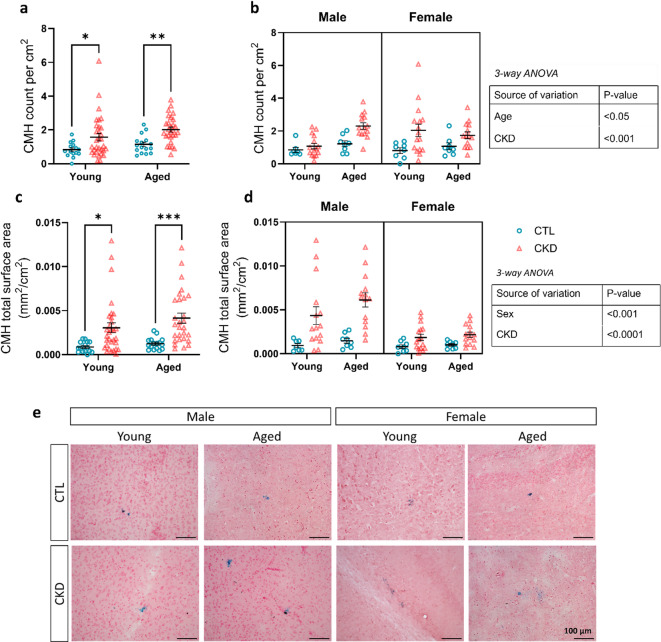
Effects of sex and age on cerebral microhemorrhage (CMH) burden in CKD mice. CMH were quantified on Prussian blue histology. Panels (**a**) and (**c**) show aggregate male and female data for CMH count and total surface area. Panels (**b**) and (**d**) demonstrate impact of age, sex and CKD status (3-way ANOVA). Panel (**e**) Representative CMH images on Prussian blue histology from CTL and CKD animals (20x objective, scale bar = 100 μm). Data presented as mean ± SEM. *p < 0.05, **p < 0.01, ***p < 0.001.

To investigate the influence of sex and age on CMH formation in CKD and CTL mice, brain hemispheres were analyzed for CMH number, total surface area, and average CMH size. CMH counts and cumulative surface area were normalized to total brain surface area. When considering aggregate male and female data, normalized CMH counts and surface area were significantly higher in both young and aged CKD mice compared with CTL counterparts (Fig. 1a, c).

When stratified by sex, increased CMH number was observed in aged CKD males compared with their young counterparts (*p* < 0.01). In females, the count was higher in the young CKD group compared with young CTL females (*p* < 0.05) (Fig. 1b). In both young and aged CKD models, male mice had a larger CMH surface area compared with CTL animals and female mice (*p* < 0.05) (Fig. 1d). Three-way ANOVA revealed that CKD significantly influenced CMH count (*p* < 0.001), total surface area (*p* < 0.0001), and average CMH size (*p* < 0.01). Sex significantly modified CMH total surface area (*p* < 0.001) and CMH size (*p* < 0.01), while age significantly influenced CMH counts (*p* < 0.05). Significant interactions were found for age x CKD on CMH count (*p* < 0.05), while sex x CKD influenced CMH surface area (*p* < 0.01) as well as CMH size (*p* < 0.05).

### Uremic toxin levels vary by sex and age in CKD mice

**Fig. 2:**
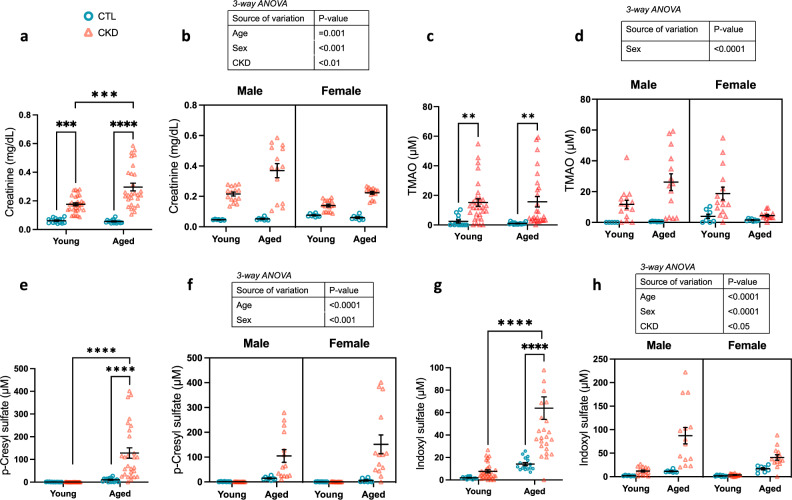
Serum creatinine and uremic toxin levels vary by age and sex in the mouse adenine-CKD model. Panels (**a**), (**c**), (**e**) and (**g**) show aggregate male and female data. Panels (**b**), (**d**), (**f**) and (**h**) demonstrate impact of sex, CKD status and age on serum levels of the toxins of interest. Data presented as mean ± SEM. TMAO = trimethylamine N-oxide. **p < 0.01, ***p < 0.001, ****p < 0.0001.

To investigate the effect of altered uremic toxin levels on CMH formation, we first assessed the impact of age and sex on uremic toxin levels in CKD mice. Serum creatinine (marker of kidney function) as well as levels of serum gut-derived uremic toxins (pCS, IS, and TMAO) were measured. Data are summarized in Table 1 and presented in Fig. 2. Serum creatinine was significantly increased with CKD in both young (*p* < 0.0001) and aged (*p* < 0.0001) mice. Aged CKD mice had higher creatinine levels compared with young CKD counterparts (*p* < 0.0001) (Fig. 2a). When stratified by sex, aged CKD males had significantly higher creatinine levels compared with aged females (*p* < 0.0001). Three-way ANOVA revealed significant effects of age (*p* = 0.001), sex (*p* < 0.001) and CKD status (*p* < 0.01) on serum creatinine (Fig. 2b). There was significant interaction between sex and age (*p* < 0.001), as well as sex and CKD (*p* < 0.001).

When considering aggregate male and female data, levels of the gut-derived uremic toxins TMAO, pCS and IS were significantly higher in aged CKD mice compared with aged CTL (*p* < 0.01, *p* < 0.0001 and *p* < 0.0001, respectively) (Fig. 2c,e,g). Conversely, among young CKD mice, only TMAO was significantly increased compared with young CTL animals (*p* < 0.01). In both sexes, pCS and IS were higher in aged than in young CKD mice. TMAO was higher in aged vs. young CKD mice only in males (Fig. 2d,f,h). CKD status had a significant impact on IS levels in young and aged animals. Male aged CKD animals had significantly higher levels of TMAO (*p* < 0.001) and IS (*p* < 0.001) compared with female aged CKD counterparts.

**Table 1:** Parameters at study termination in control (CTL) and chronic kidney disease (CKD) animals.

	Male				Female			
	Young		Aged		Young		Aged	
	CTL *n* = 7	CKD *n* = 15	CTL *n* = 8	CKD *n* = 14	CTL *n* = 8	CKD *n* = 16	CTL *n* = 8	CKD *n* = 14
Serum creatinine (mg/dL)	0.05 ± 0.001	0.22 ± 0.01^a^	0.05 ± 0.003	0.37 ± 0.05^ab^	0.08 ± 0.003	0.14 ± 0.01	0.06 ± 0.01	0.22 ± 0.01^abc^
TMAO (µM)	Not detected	11.7 ± 2.7	0.6 ± 0.1	26.1 ± 5.4^ab^	3.9 ± 1.4	18.7 ± 4.2	1.5 ± 0.3	4.5 ± 0.7^bc^
p-Cresyl Sulfate (µM)	1.1 ± 0.2	0.9 ± 0.1	14.6 ± 2.2	104.6 ± 25.7^b^	0.3 ± 0.2	0.5 ± 0.2	5.9 ± 2.5	151.3 ± 37.9^ab^
Indoxyl Sulfate (µM)	2.0 ± 0.3	12.0 ± 2.1	11.4 ± 1.0	87.1 ± 17.5^ab^	1.8 ± 0.5	3.7 ± 0.6	16.9 ± 2.3	40.8 ± 5.7^bc^
Normalized CMH count per cm^2^	0.85 ± 0.15	1.08 ± 0.17	1.22 ± 0.18	2.30 ± 0.20^b^	0.81 ± 0.17	2.03 ± 0.38^a^	1.06 ± 0.20	1.74 ± 0.21
Normalized CMH area (mm^2^/cm^2^)	0.0009 ± 0.0002	0.0040 ± 0.0010^a^	0.0015 ± 0.0003	0.0060 ± 0.0010^a^	0.0008 ± 0.0002	0.0020 ± 0.0004^c^	0.0010 ± 0.0001	0.0020 ± 0.00030^c^
Average CMH size (mm^2^)	0.0010 ± 0.0002	0.0040 ± 0.0010^a^	0.0020 ± 0.0005	0.0030 ± 0.0005	0.0010 ± 0.0003	0.0009 ± 0.0001^c^	0.0010 ± 0.0002	0.0015 ± 0.0003
Shannon diversity index	4.690 ± 0.05	3.912 ± 0.17	4.580 ± 0.08	4.433 ± 0.07	4.806 ± 0.07	4.595 ± 0.07	4.586 ± 0.10	4.489 ± 0.08

Data presented as mean ± SEM. CMH = cerebral microhemorrhages; TMAO = trimethylamine N-oxide. a: *p* < 0.05 compared with age- and sex- matched CTL; b: *p* < 0.05 compared with young sex-matched CKD; c: *p* < 0.05 sex difference compared with age-matched CKD males.

### Effect of age on toxin-CMH associations

**Fig. 3:**
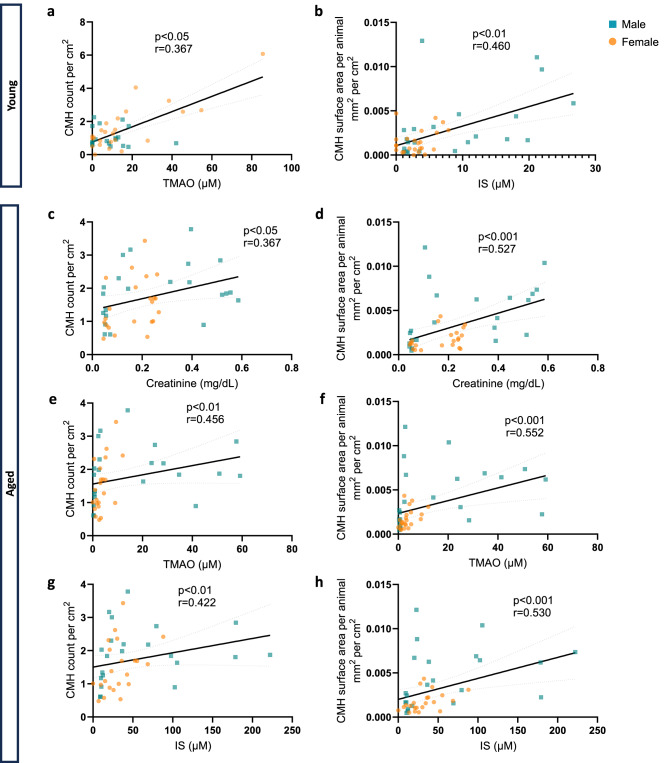
Aged mice show stronger associations between uremic toxin levels and cerebral microhemorrhage (CMH) burden. Both young and aged mice demonstrated positive correlations between trimethylamine N-oxide (TMAO) and normalized CMH counts (**a**,**e**), as well as between indoxyl sulfate (IS) and CMH surface area (**b**,**h**). The correlations were stronger in aged mice. Additional correlations were observed in aged mice between serum creatinine, TMAO and IS with CMH parameters (**c**–**h**).

To investigate the role of age on the relationships between uremic toxin levels and CMH formation, the dataset was analyzed using Spearman’s correlation coefficient and stratified by age. In young mice, CMH counts were positively correlated with TMAO levels (*p* < 0.05) (Fig. 3a), and CMH total surface area correlated with IS (*p* < 0.01) (Fig. 3b). These associations were stronger in aged animals (Figs. 3e and h). Aged mice demonstrated additional significant correlations between CMH burden with serum creatinine, TMAO and IS (Fig. 3). pCS did not have a significant relationship with CMH burden in young or aged animals.

### Sex-specific alterations in gut microbiota in aged CKD mice

**Fig. 4:**
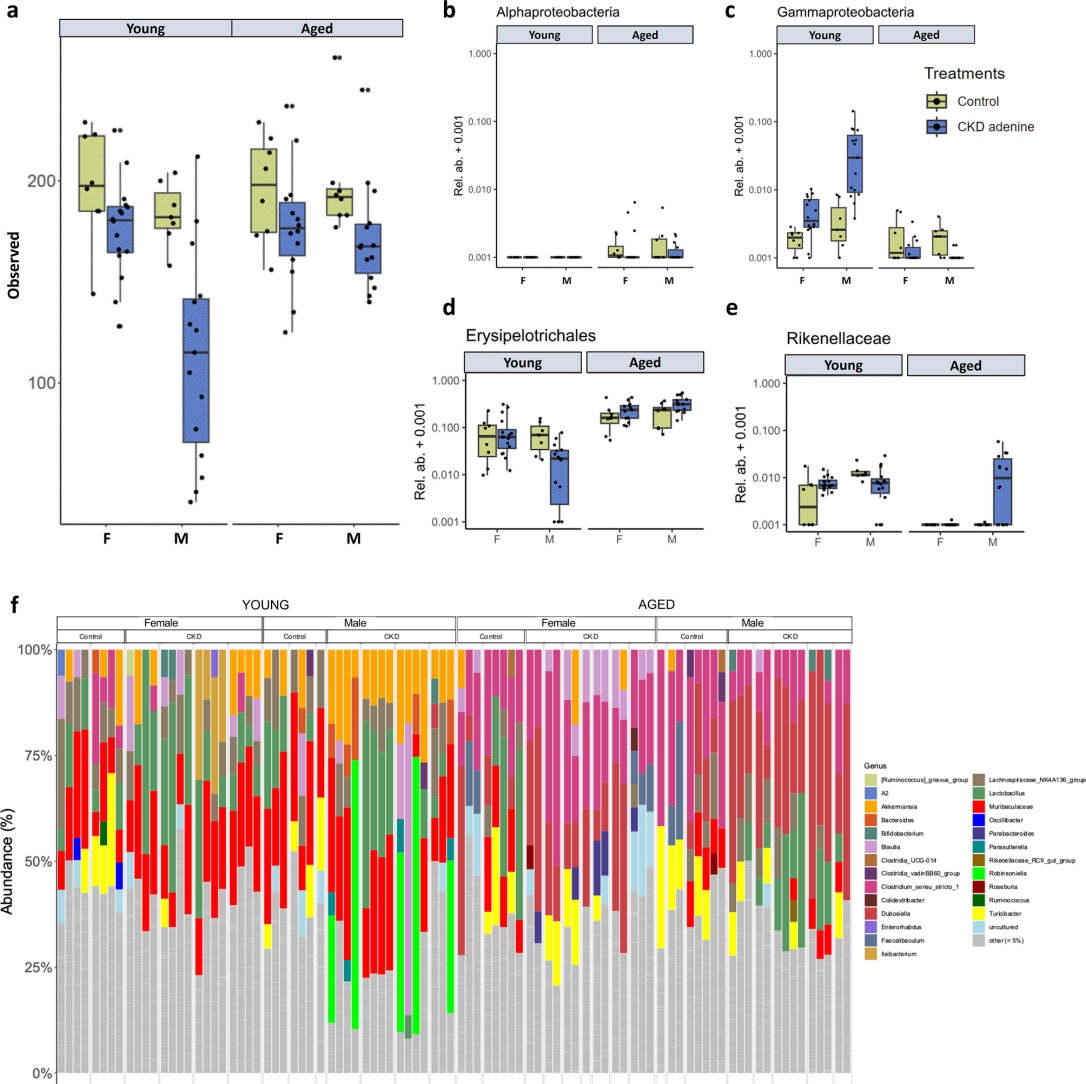
Gut dysbiosis in young vs. aged CKD mice, in both sexes. Altered richness of gut microbiome (**a**), and relative abundance of proteolytic (**b**,**c**) and saccharolytic (**d**,**e**) bacteria groups in CKD vs. control mice. The most abundant bacterial genera present in each animal’s gut microbiome is summarized in panel f. F = females, M = males.

To examine the influence of sex, age, and CKD status on gut-microbiota, 16S rRNA sequencing was done from fecal pellets (Fig. 4). In general, CKD groups had lower gut microbial richness than sex- and age-matched controls, and young male CKD mice had the lowest richness (Fig. 4a). On beta diversity analysis, age had the biggest effect on the microbiome and explained 29.2% of the variance; sex explained 3.9% and CKD status explained 2.7%. Relative abundance of the proteolytic bacterium *Gammaproteobacteria* was higher in young male and female CKD mice compared with their control counterparts; conversely, proteolytic *Alphaproteobacteria* and *Gammaproteobacteria* were less abundant in aged CKD mice in both sexes compared with their control counterparts (Fig. 4b and c). The relative abundance of saccharolytic bacteria was similarly modified by both sex and age. Aged mice exhibited a higher relative abundance of *Erysipelotrichales* compared with young mice. Among young CKD males, the abundance of *Erysipelotrichales* was notably decreased (Fig. 4d). In aged female control and CKD mice, the abundance of another saccharolytic bacteria, *Rikenellaceae*, was decreased compared with young females. Among aged groups, aged CKD males were noted to have the highest abundance of *Rikenellaceae* (Fig. 4e).

The most abundant bacterial genera present in each animal’s gut microbiome is summarized in Fig. 4f. When comparing young vs. aged, *Muribaculaceae* and *Lactobacillus* were prevalent in the microbiome from young animals, whereas *Clostridium* and *Dubosiella* were highly abundant in aged animals. When analyzing for CKD effects on the microbiome, young CKD males consistently showed increased abundance of *Akkermansia*. Cage effects were evident in young CKD females, with one cage (*n* = 4) demonstrating increased *Ileibacterium* and another cage (*n* = 4) with increased *Akkermansia*. In aged CKD vs. control mice, CKD did not exert a consistent effect on microbial populations.

## Discussion

In this study of young and aged mice with adenine-induced CKD compared with CTL mice, male aged CKD animals had the highest CMH count and total CMH surface area. Three-way ANOVA confirmed that CKD significantly influenced CMH count, total surface area and average CMH size. The interaction of age with CKD status significantly influenced CMH count, whereas the interaction of sex with CKD status had a significant impact on the CMH total surface area per brain and CMH size. Associations between uremic toxins and CMH burden were stronger in aged than in young mice. CKD groups had lower gut microbial richness than sex- and age-matched controls. However, age had the biggest effect on the gut microbiome and explained 29.2% of the variance.

Consistent with our prior report in aged CKD animals^11^CMH counts were higher in aged male than in female CKD mice and CMH burden was significantly associated with the degree of adenine-induced kidney injury. Our group previously reported that aging increases the brain’s vulnerability to inflammation-induced CMH in mice, in part due to microglial activation^13^. Activated microglia release inflammatory cytokines with induction of matrix metalloproteinases (MMP-2 and MMP-9) and oxidative stress, which promote blood-brain barrier disruption and CMH formation^14^. We also observed that male CKD mice exhibited a higher CMH surface area compared with female counterparts. This sex difference is partly explained by more severe adenine-induced CKD (higher serum creatinine) in males (Fig. 2; Table 1). Further, males have heightened neuroinflammatory responses compared with females: Rossetti et al. reported that LPS exposure in male mice induced higher levels of interleukin-1β and tumor necrosis factor-α in the hippocampus and frontal cortex compared female animals^15^.

Hypertension is a known risk factor for brain microhemorrhages^10^ and a limitation of the current study is that blood pressure was not measured. However, we and others have previously reported that blood pressure is not elevated in the adenine CKD model^11,16,17^. Therefore, blood pressure is unlikely to be a major contributing factor to CMH formation in the current mouse cohorts.

Serum creatinine, IS and pCS were higher in aged CKD mice than in young CKD counterparts. Surprisingly, although pCS was markedly elevated in aged CKD animals and prior evidence suggests that pCS exposure induces vascular endothelium injury^18,19 ^, this gut-derived toxin did not correlate with CMH burden in our study. This suggests that pCS effects in the brain may be less important on microvascular pathways, and more relevant in neuronal cell apoptosis, oxidative stress, and neuroinflammation^20^. Conversely, TMAO was correlated with CMH counts and surface area, and IS was associated with CMH surface area, consistent with their role as vascular toxins^21,22^.

TMAO showed a different pattern in females, whereby levels were highest in young female CKD mice. In CKD, both decreased renal TMAO clearance and increased hepatic flavin monoxoygenases (which generate TMAO) contribute to increased serum TMAO^23^. Studies in non-CKD mice have attributed higher TMAO in females than males to higher hepatic expression of flavin monooxygenase-3 (FMO3)^7^. Paradoxically, upregulation of liver FMO3 is associated with longevity in experimental models^24^. Further studies are needed to elucidate FMO3 physiology in the setting of aging combined with CKD. Regardless, our study noted strong associations between creatinine and TMAO levels with higher CMH burden (Fig. 3), emphasizing that degree of kidney dysfunction is an important risk factor for microvascular disease^10,11,25^.

Gut dysbiosis in CKD has been described in dialysis patients and in pre-clinical CKD models, whereby expansion of bacteria that express urease, uricase, and p-cresyl- and indole-forming enzymes is concurrent with depletion of beneficial bacteria that produce short-chain fatty acids^3,26,27^. To our knowledge, our study is the first to explore gut dysbiosis in aged CKD mice. The 16S rRNA sequencing data highlighted age as the major modifier of gut microbial populations (age explained 29.2% of the variance; sex explained 3.9% and CKD status explained 2.7% of the variance). When looking at most abundant bacterial genera (Fig. 4f), aged CKD animals had a similar gut microbiome to aged controls; i.e., the microbiome was resistant to changes exerted by the CKD milieu. Of note, young CKD males had increased gut *Akkermansia*, a beneficial microbe which metabolizes mucin to acetate and propionate, short-chain fatty acids that are nutrients for the host’s enterocytes with downstream regulation of adipose tissue and glucose metabolism^28–31^. This effect, presumably a defense mechanism in the setting of gut dysbiosis, was not seen in aged CKD animals. Further, aged CKD animals did not manifest expansion of proteolytic bacteria such as *Alphaproteobacteria* and *Gammaproteobacteria* which was observed in young CKD animals. More work is needed to reconcile this finding with the lower TMAO levels observed in aged female CKD mice, since proteolytic bacteria contribute to the production of gut-derived uremic toxins^3^.

**Fig. 5:**
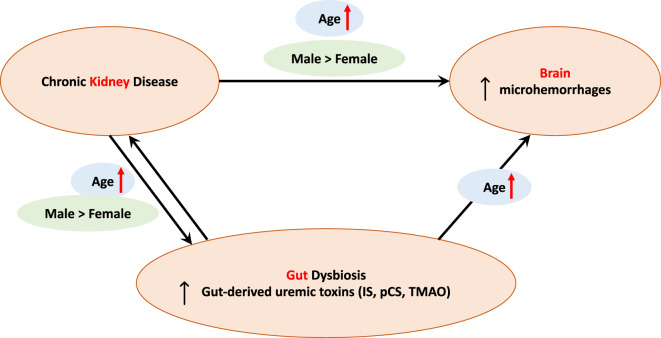
Age and sex modify the kidney-gut-brain axis. Age and sex are key modulators of gut dysbiosis and uremic toxin production in chronic kidney disease, which contribute to cerebral microhemorrhage (CMH) formation. In addition, age and sex independently impact CMH formation.

In summary, this study of the kidney-gut-brain axis noted significant effects of age, sex and CKD status on CMH burden. Aged male CKD mice had the highest CMH count and CMH total surface area, concurrent with higher serum creatinine (indicating more severe kidney dysfunction) and higher TMAO levels compared with other CKD groups. Alterations in the gut microbiota were predominantly influenced by age, and the gut-derived toxins TMAO and IS (but not pCS) were correlated with CMH outcomes. Our findings highlight the complex interplay of CKD, sex and age on gut dysbiosis and brain microvascular health (Fig. 5).

## Materials and methods

### Animal model

**Fig. 6:**
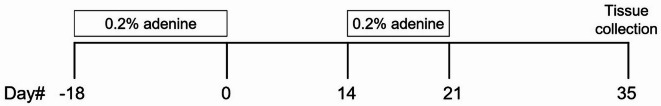
Adenine CKD model timeline. Young and aged mice were fed diet containing 0.2% adenine for 18 days to induce tubulointerstitial nephritis. Mice were then fed regular chow for 14 days, followed by re-expose to the adenine diet for 7 days. Mice were sacrificed 5 weeks after CKD induction.

All animal studies were approved by the Animal Research Committee, University of California, Irvine (UCI) under protocol AUP-21-157. Animals were maintained at the UCI vivarium in accordance with the policies instituted by the American Association for Accreditation of Laboratory Animal Care. We confirm that all methods used in this study are reported in accordance with the ARRIVE guidelines for the reporting of animal experiments. Every aspect of the experiment design, including ethical approval, animal care, randomization, blinding, and statistical analysis, has been documented and presented in a manner consistent with the guides to ensure transparency, reproducibility, and the ethical use of animals in research.

Tubulointerstitial nephritis was induced via nephrotoxic adenine diet in adult C57BL/6J mice (Fig. 6). Young mice (10–12 weeks old, Jackson Laboratories, Bar Harbor, ME)) and aged mice (16 months old, National Institute on Aging aged rodent colony) were fed a diet containing 0.2% adenine for 18 days (Dyet# 611732, Dyets Inc., Bethlehem, PA), placed back on regular chow for 2 weeks, and then re-exposed to adenine diet for 1 week to maintain CKD^11,16^. Control (CTL) age-matched animals were maintained on regular feed for the duration of the experiment. Studies were done in both male and female animals.

### Tissue collection

At study termination, the chest cavity was opened, and blood was collected via cardiac puncture under inhaled isoflurane anesthesia and centrifuged for serum collection (BD microtainer with clot activator, catalog# 2675185, Thermo Fisher Scientific). Perfusion was done for 5 min with ice-cold phosphate-buffered saline (PBS) at a flow rate of 7–8 mL/min via a 26-gauge needle in the left ventricle, followed by brain collection. The left hemisphere was snap frozen and stored at -80 °C for future molecular analysis. The right brain hemisphere was fixed in 4% paraformaldehyde at 4 °C for 24 h, then cryoprotected in a sucrose gradient at 4 °C (15% sucrose for 24 h, followed by 30% sucrose for another 24 h). The fixed tissues were subsequently stored at -80 °C until time of sectioning for histology. Fecal pellets were collected from the descending colon, snap frozen and stored at -80 °C for gut microbiota analysis.

### Stool microbiome sequencing

Two stool pellets per animal were transported in 500 µL of RNA/DNA Shield™ (Catalog# R1101, (Zymo Research, Irvine, CA) to the Zymo Research laboratory (Irvine, CA). Extraction of microbial DNA was performed with a bead-beating step using the ZymoBIOMICS^®^-96 MagBead DNA Kit. DNA samples were prepared for targeted sequencing with the Quick-16 S™ NGS Library Prep Kit with custom designed Primer set V3-V4 (Zymo Research). Control Samples: The ZymoBIOMICS^®^ Microbial Community Standard was used as a positive control for each DNA extraction, and a negative control (i.e., blank extraction control) was included to assess the level of bioburden carried by the wet-lab process. The sequencing library PCR products were quantified with qPCR fluorescence readings, pooled based on equal molarity, and cleaned up with the Select-a-Size DNA Clean & Concentrator™ (Zymo Research), then quantified with TapeStation^®^ (Agilent Technologies, Santa Clara, CA) and Qubit^®^ (Thermo Fisher Scientific, Waltham, WA). The final library was sequenced on Illumina^®^ MiSeq™ with a v3 reagent kit (600 cycles). The sequencing was performed in 10% PhiX spike-in. At the UCI Microbiome Center, the sequence data were quality checked and demultiplexed in QIIME 2^32^ with rarefaction at 4300 sequences/sample, and was clustered into 100% Operational Taxonomic Units (OTUs, a.k.a. Exact Sequence Variants, ESVs) and analyzed for alpha- and beta-diversity metrics within the Vegan package in R^33^.

### Quantification of serum uremic toxins

Serum creatinine was measured using capillary electrophoresis at the O’Brien Kidney Research Core Center (UT Southwestern, Dallas, TX). Serum levels of total (free and protein-bound) gut-derived uremic toxins were quantified using Xevo Tandem Quadrupole Mass Spectrometer (TQ Abs-MS instrument, Waters Quattro Premier XE equipped with UPLC). A 20 µL aliquot of serum was treated with 200 µL of acetonitrile with 0.1% formic acid and internal standards (hydrochlorothiazide 2 µg/mL and salbutamol 5 ng/mL) for protein precipitation. The mixture was vortexed and centrifuged, and the supernatant was evaporated to dryness. The dried extract was reconstituted with 100 µL of 25% acetonitrile. Standards and prepared samples were injected (10 µL) into the TQ Abs-MS instrument. For indoxyl sulfate (catalog# I3875, Sigma) and p-cresyl sulfate (p-tolyl sulfate, catalog# P2091, Fisher Scientific), the internal standard used was hydrochlorothiazide (catalog# 5001437615, Fisher Scientific) and analysis was performed in negative ionization mode with buffer A2 (10 mM ammonium formate + 0.05% formic acid) and B2 (100% methanol). For TMAO (catalog# 317594, Sigma), the internal standard used was salbutamol (catalog# S8260, Sigma) and analysis was performed in positive ionization mode. Standard curves were generated in 25% acetonitrile with hydrochlorothiazide 4 $\:{\upmu\:}$g/mL and salbutamol 10 ng/mL (highest standard 10,000 ng/mL). The buffer of the positive mode is A1 (0.2% acetic acid, 2% acetonitrile in water), and B1 (100% acetonitrile + 0.2% acetic acid). The transition (*m/z*) values are indoxyl sulfate 211.97→80.36, p-cresyl sulfate 186.94→107.30, TMAO 76→59, hydrochlorothiazide 296.96→270.08, salbutamol 240→148.

### Brain microhemorrhage histology

One brain hemisphere was sectioned into 20-µm coronal sections. Sections were collected every 200 μm and stained with Prussian blue to detect hemosiderin (a marker of iron deposition within CMH)^34–36^. Nuclear fast red was used as a counterstain. Digitized images were analyzed using ImageJ v1.54d (NIH, Bethesda, MD, USA, https://imagej.nih.gov/ij/) by an observer blinded to the experimental groups. For each mouse, CMH number and total CMH surface area (mm^2^) were normalized to total brain surface area (cm^2^). Average CMH size (mm^2^) was calculated per animal.

### Statistical analysis

Data were analyzed using GraphPad Prism 9 (GraphPad Software, Lo Jolla, CA) and R version 4.4.2 (R Core Team, Vienna, Austria). ROUT with Q = 1% was used to exclude outliers. Results are presented as mean ± SEM. Normality tests were performed using D’Agostino-Pearson. Šídák’s multiple comparisons test was used to determine age, sex and CKD status effects via 3-way ANOVA. Correlation between continuous variables was determined using Spearman’s correlation coefficient (r). Gut microbial alpha- and beta-diversity metrics were analyzed within the Vegan package in R (Oksanen). Shannon diversity of gut microbiota was analyzed using a linear mixed effects model (nlme package in R) after accounting for cage housing (adjustment for animals housed in the same cage). *P* < 0.05 was considered statistically significant. Beta diversity significance was tested using PERMANOVA with adonis as part of the vegan package in R. To check for dispersion differences, beta disper was used as a part of the vegan package in R.

## Data Availability

All raw data that supports the findings of this study are available in Dryad datasets (DOI: 10.5061/dryad.95x69p8x2). All sequencing data generated in this study have been deposited in the NCBI Sequence Read Archive under BioProject ID PRJNA954040. A complete list of individual accession numbers is provided in Dryad datasets.

## References

[1] Durack, J. & Lynch, S. V. The gut microbiome: relationships with disease and opportunities for therapy. *J. Exp. Med.* **216**, 20–40. 10.1084/jem.20180448 (2019). 30322864 10.1084/jem.20180448 PMC6314516

[2] Vaziri, N. D. et al. Chronic kidney disease alters intestinal microbial flora. *Kidney Int.* **83**, 308–315. 10.1038/ki.2012.345 (2013). 22992469 10.1038/ki.2012.345

[3] Lau, W. L., Savoj, J., Nakata, M. B. & Vaziri, N. D. Altered Microbiome in chronic kidney disease: systemic effects of gut-derived uremic toxins. *Clin. Sci. (Lond)*. **132**, 509–522. 10.1042/CS20171107 (2018). 29523750 10.1042/CS20171107

[4] Yang, T., Richards, E. M., Pepine, C. J. & Raizada, M. K. The gut microbiota and the brain-gut-kidney axis in hypertension and chronic kidney disease. *Nat. Rev. Nephrol.* **14**, 442–456. 10.1038/s41581-018-0018-2 (2018). 29760448 10.1038/s41581-018-0018-2 PMC6385605

[5] Noce, A. et al. Link between gut microbiota dysbiosis and chronic kidney disease. *Eur. Rev. Med. Pharmacol. Sci.* **26**, 2057–2074. 10.26355/eurrev_202203_28354 (2022). 35363356 10.26355/eurrev_202203_28354

[6] Wehedy, E., Shatat, I. F. & Al Khodor, S. The human microbiome in chronic kidney disease: A double-edged sword. *Front. Med. (Lausanne)*. **8**, 790783. 10.3389/fmed.2021.790783 (2021). 35111779 10.3389/fmed.2021.790783 PMC8801809

[7] Wang, Z. et al. Gut flora metabolism of phosphatidylcholine promotes cardiovascular disease. *Nature* **472**, 57–63. 10.1038/nature09922 (2011). 21475195 10.1038/nature09922 PMC3086762

[8] Fernandez-Prado, R. et al. Nutrients turned into toxins: microbiota modulation of nutrient properties in chronic kidney disease. *Nutrients* **9**10.3390/nu9050489 (2017). 10.3390/nu9050489 PMC5452219 28498348

[9] Jazani, N. H., Savoj, J., Lustgarten, M., Lau, W. L. & Vaziri, N. D. Impact of gut dysbiosis on neurohormonal pathways in chronic kidney disease. *Diseases* **7**10.3390/diseases7010021 (2019). 10.3390/diseases7010021 PMC6473882 30781823

[10] Lau, W. L., Huisa, B. N. & Fisher, M. The cerebrovascular-chronic kidney disease connection: Perspectives and mechanisms. *Transl Stroke Res.* **8**, 67–76. 10.1007/s12975-016-0499-x (2017). 27628245 10.1007/s12975-016-0499-x PMC5241336

[11] Fang, C. et al. Chronic kidney disease promotes cerebral microhemorrhage formation. *J. Neuroinflammation*. **20**10.1186/s12974-023-02703-2 (2023). 10.1186/s12974-023-02703-2 PMC9960195 36841828

[12] Lau, W. L. et al. Cystatin C, cognition, and brain MRI findings in 90+-year-olds. *Neurobiol. Aging*. **93**, 78–84. 10.1016/j.neurobiolaging.2020.04.022 (2020). 32473464 10.1016/j.neurobiolaging.2020.04.022 PMC7307913

[13] Sumbria, R. K. et al. Aging exacerbates development of cerebral microbleeds in a mouse model. *J. Neuroinflammation*. **15**. 10.1186/s12974-018-1092-x (2018). 10.1186/s12974-018-1092-x PMC5840821 29510725

[14] da Fonseca, A. C. et al. The impact of microglial activation on blood-brain barrier in brain diseases. *Front. Cell. Neurosci.* **8**, 362. 10.3389/fncel.2014.00362 (2014). 25404894 10.3389/fncel.2014.00362 PMC4217497

[15] Rossetti, A. C. et al. Differential neuroinflammatory response in male and female mice: A role for BDNF. *Front. Mol. Neurosci.* **12**, 166. 10.3389/fnmol.2019.00166 (2019). 31379496 10.3389/fnmol.2019.00166 PMC6658805

[16] Lau, W. L. et al. Chronic kidney disease increases cerebral microbleeds in mouse and man. *Transl Stroke Res.* **11**, 122–134. 10.1007/s12975-019-00698-8 (2020). 31055735 10.1007/s12975-019-00698-8 PMC6957561

[17] Mori-Kawabe, M., Yasuda, Y., Ito, M. & Matsuo, S. Reduction of NO-mediated relaxing effects in the thoracic aorta in an experimental chronic kidney disease mouse model. *J. Atheroscler Thromb.* **22**, 845–853. 10.5551/jat.28191 (2015). 25740549 10.5551/jat.28191

[18] Tang, W. H. et al. Gut microbiota-dependent trimethylamine N-Oxide (TMAO) pathway contributes to both development of renal insufficiency and mortality risk in chronic kidney disease. *Circ. Res.* **116**, 448–455. 10.1161/CIRCRESAHA.116.305360 (2015). 25599331 10.1161/CIRCRESAHA.116.305360 PMC4312512

[19] Shah, S. N. et al. Cerebrovascular damage caused by the gut microbe/host co-metabolite. *Gut Microbes*. **16**, 2431651. 10.1080/19490976.2024.2431651 (2024). 39582109 10.1080/19490976.2024.2431651 PMC11591591

[20] Sun, C. Y. et al. p-Cresol sulfate caused behavior disorders and neurodegeneration in mice with unilateral nephrectomy involving oxidative stress and neuroinflammation. *Int. J. Mol. Sci.* **21**10.3390/ijms21186687 (2020). 10.3390/ijms21186687 PMC7555291 32932690

[21] Barreto, F. C. et al. Serum indoxyl sulfate is associated with vascular disease and mortality in chronic kidney disease patients. *Clin. J. Am. Soc. Nephrol.* **4**, 1551–1558. 10.2215/CJN.03980609 (2009). 19696217 10.2215/CJN.03980609 PMC2758258

[22] Dolkar, P. et al. Trimethylamine-N-oxide and cerebral stroke risk: A review. *Neurobiol. Dis.* **192**, 106423. 10.1016/j.nbd.2024.106423 (2024). 38286388 10.1016/j.nbd.2024.106423

[23] Johnson, C., Prokopienko, A. J., West, R. E., Nolin, T. D. & Stubbs, J. R. Decreased kidney function is associated with enhanced hepatic flavin monooxygenase activity and increased Circulating trimethylamine. *Drug Metab. Dispos.* **46**, 1304–1309. 10.1124/dmd.118.081646 (2018). 29915157 10.1124/dmd.118.081646

[24] Rossner, R., Kaeberlein, M. & Leiser, S. F. Flavin-containing monooxygenases in aging and disease: emerging roles for ancient enzymes. *J. Biol. Chem.* **292**, 11138–11146. 10.1074/jbc.R117.779678 (2017). 28515321 10.1074/jbc.R117.779678 PMC5500783

[25] Meariman, J. K., Zulli, H., Perez, A., Bajracharya, S. D. & Mohandas, R. Small vessel disease: connections between the kidney and the heart. *Am. Heart J. Plus*. **26**, 100257. 10.1016/j.ahjo.2023.100257 (2023). 38510186 10.1016/j.ahjo.2023.100257 PMC10946057

[26] Wong, J. et al. Expansion of urease- and uricase-containing, indole- and p-cresol-forming and contraction of short-chain fatty acid-producing intestinal microbiota in ESRD. *Am. J. Nephrol.* **39**, 230–237. 10.1159/000360010 (2014). 24643131 10.1159/000360010 PMC4049264

[27] Lau, W. L., Chang, Y. & Vaziri, N. D. The consequences of altered microbiota in immune-related chronic kidney disease. *Nephrol. Dial Transpl.* **36**, 1791–1798. 10.1093/ndt/gfaa087 (2021). 10.1093/ndt/gfaa087 PMC8633451 32437554

[28] Anhê, F. F. et al. A polyphenol-rich cranberry extract reverses insulin resistance and hepatic steatosis independently of body weight loss. *Mol. Metab.* **6**, 1563–1573. 10.1016/j.molmet.2017.10.003 (2017). 29107524 10.1016/j.molmet.2017.10.003 PMC5699918

[29] Everard, A. et al. Cross-talk between Akkermansia muciniphila and intestinal epithelium controls diet-induced obesity. *Proc. Natl. Acad. Sci. U S A*. **110**, 9066–9071. 10.1073/pnas.1219451110 (2013). 23671105 10.1073/pnas.1219451110 PMC3670398

[30] Derrien, M. et al. Mucin-bacterial interactions in the human oral cavity and digestive tract. *Gut Microbes*. **1**, 254–268. 10.4161/gmic.1.4.12778 (2010). 21327032 10.4161/gmic.1.4.12778 PMC3023607

[31] Lau, W. L. et al. The phosphate binder ferric citrate alters the gut microbiome in rats with chronic kidney disease. *J. Pharmacol. Exp. Ther.* **367**, 452–460. 10.1124/jpet.118.251389 (2018). 30287477 10.1124/jpet.118.251389

[32] Bolyen, E. et al. Reproducible, interactive, scalable and extensible microbiome data science using QIIME 2. *Nat. Biotechnol.* **37**, 852–857. 10.1038/s41587-019-0209-9 (2019). 31341288 10.1038/s41587-019-0209-9 PMC7015180

[33] Jari et al. Community ecology,. in *Community Ecology Package* (2007).

[34] Liu, S. et al. Comparative analysis of H&E and Prussian blue staining in a mouse model of cerebral microbleeds. *J. Histochem. Cytochem.* **62**, 767–773. 10.1369/0022155414546692 (2014). 25063000 10.1369/0022155414546692 PMC6728446

[35] Sumbria, R. K. et al. A murine model of inflammation-induced cerebral microbleeds. *J. Neuroinflammation*. **13**, 218. 10.1186/s12974-016-0693-5 (2016). 27577728 10.1186/s12974-016-0693-5 PMC5006574

[36] Sumbria, R. K. et al. Effects of phosphodiesterase 3A modulation on murine cerebral microhemorrhages. *J. Neuroinflammation*. **14**, 114. 10.1186/s12974-017-0885-7 (2017). 28583195 10.1186/s12974-017-0885-7 PMC5460510

